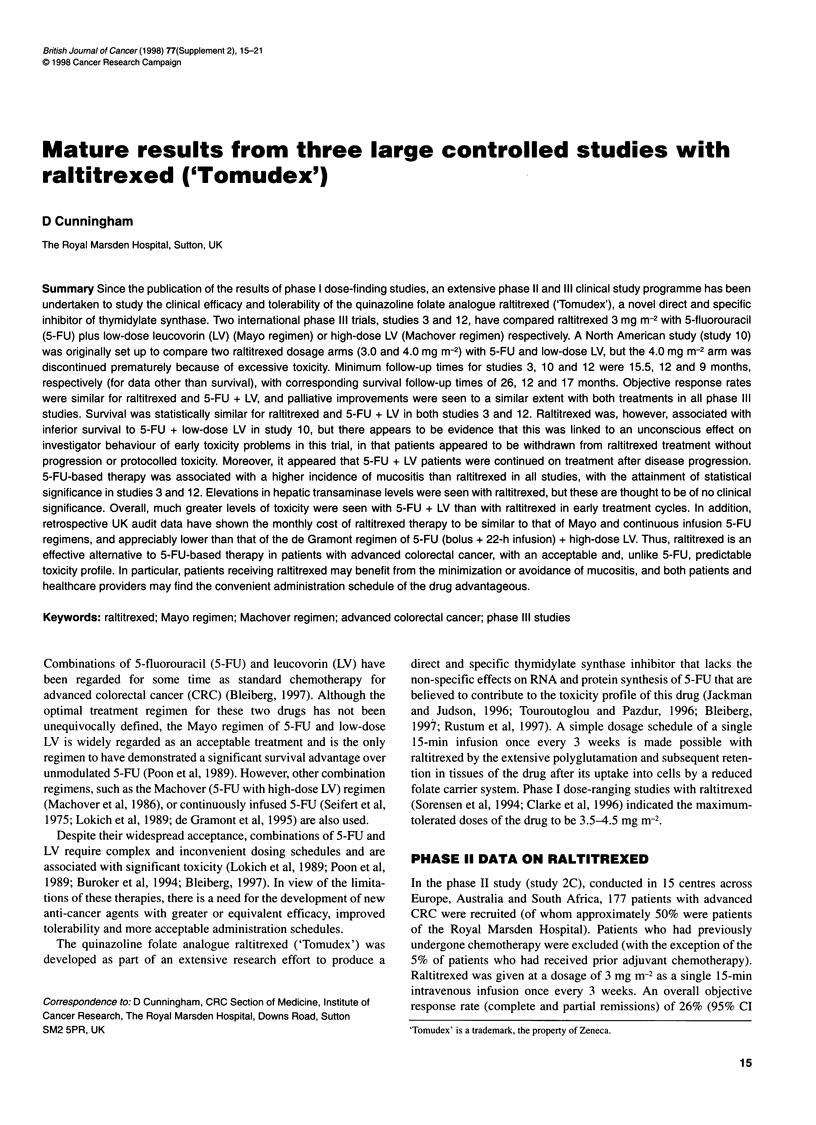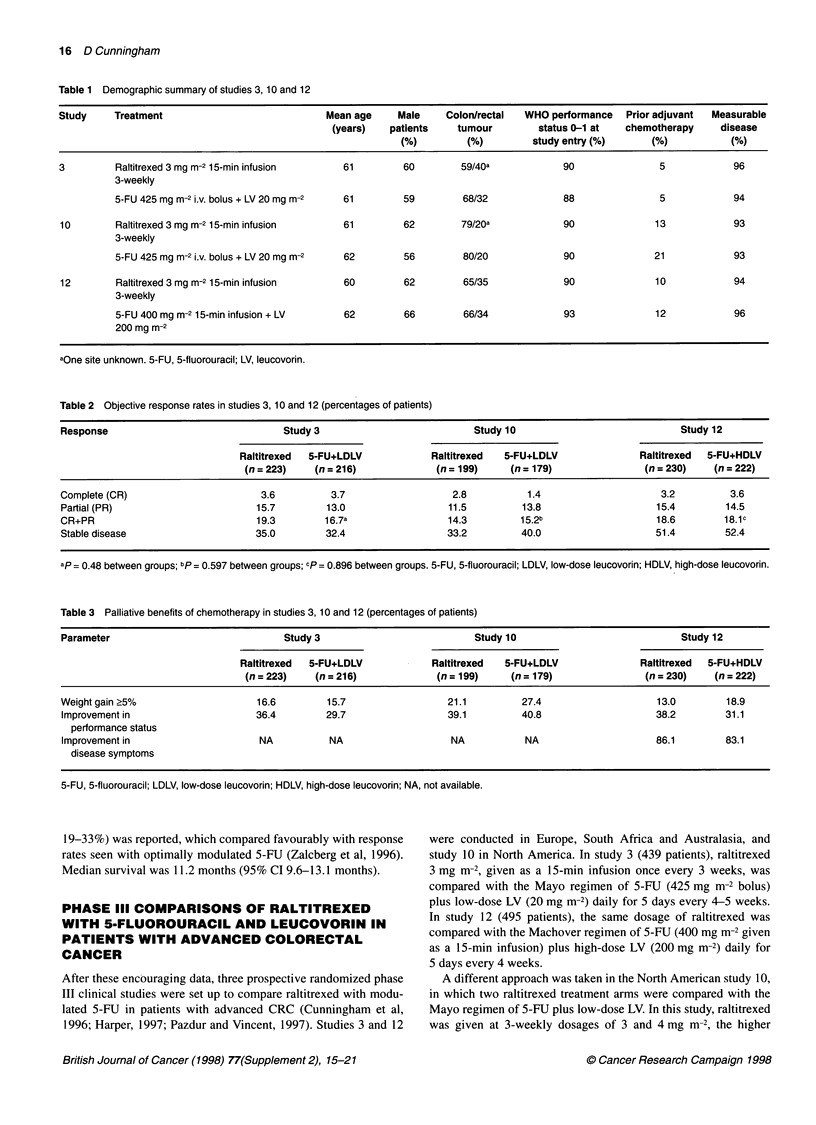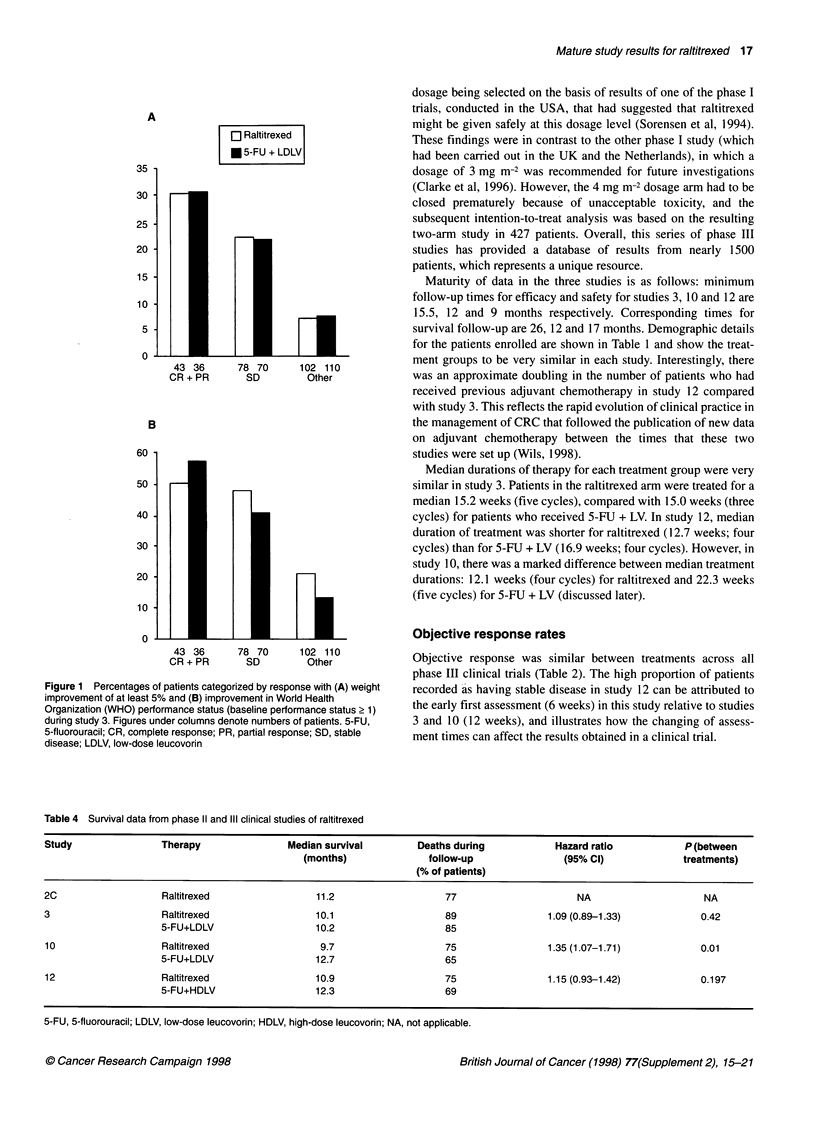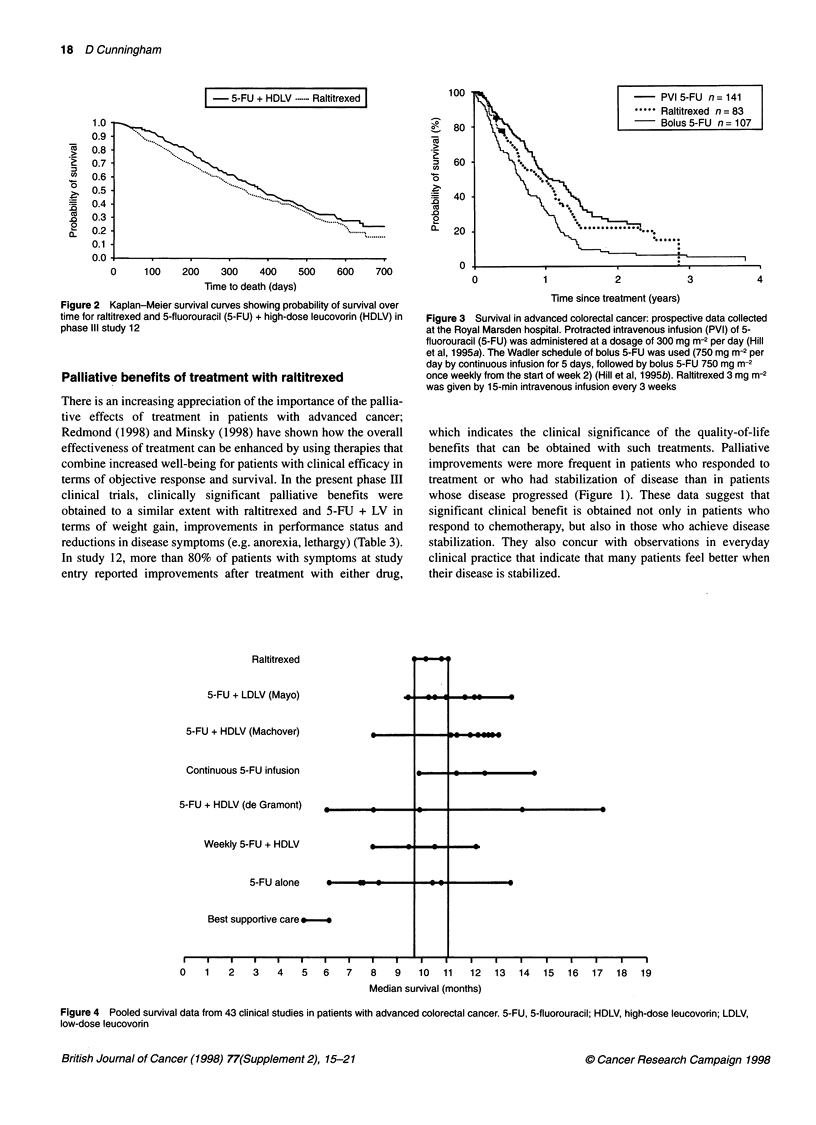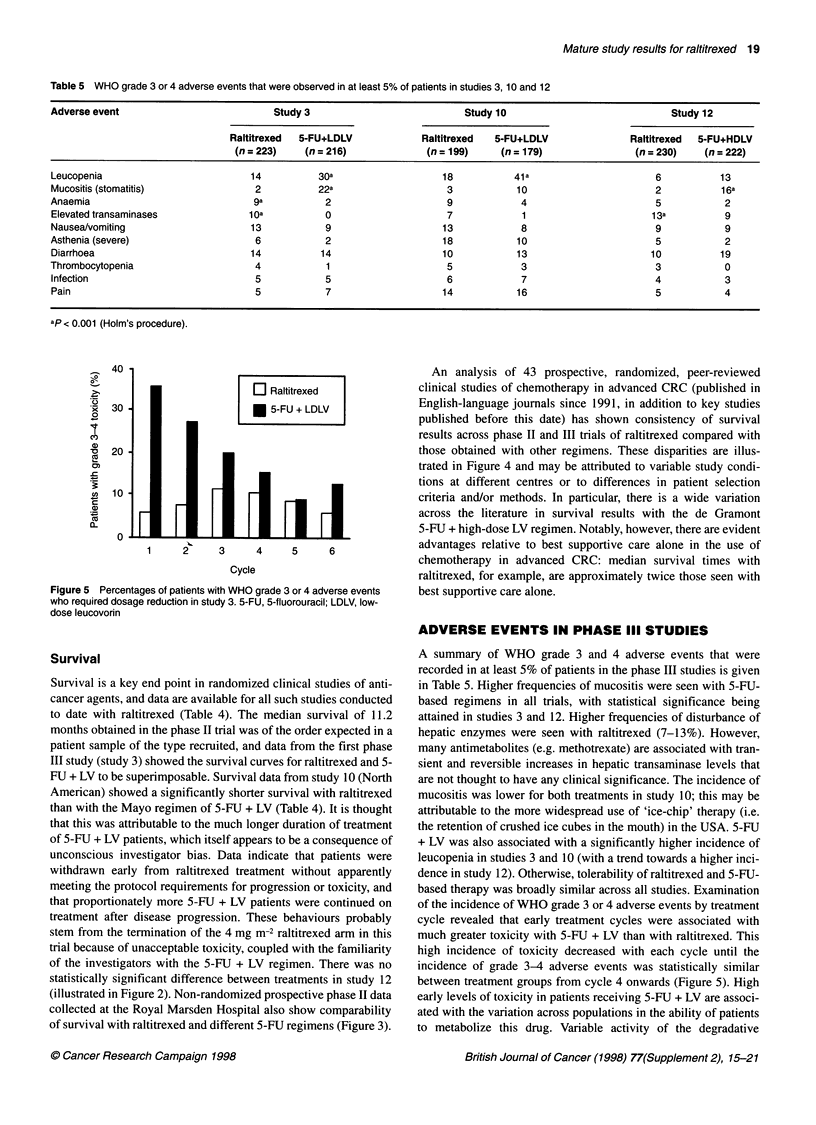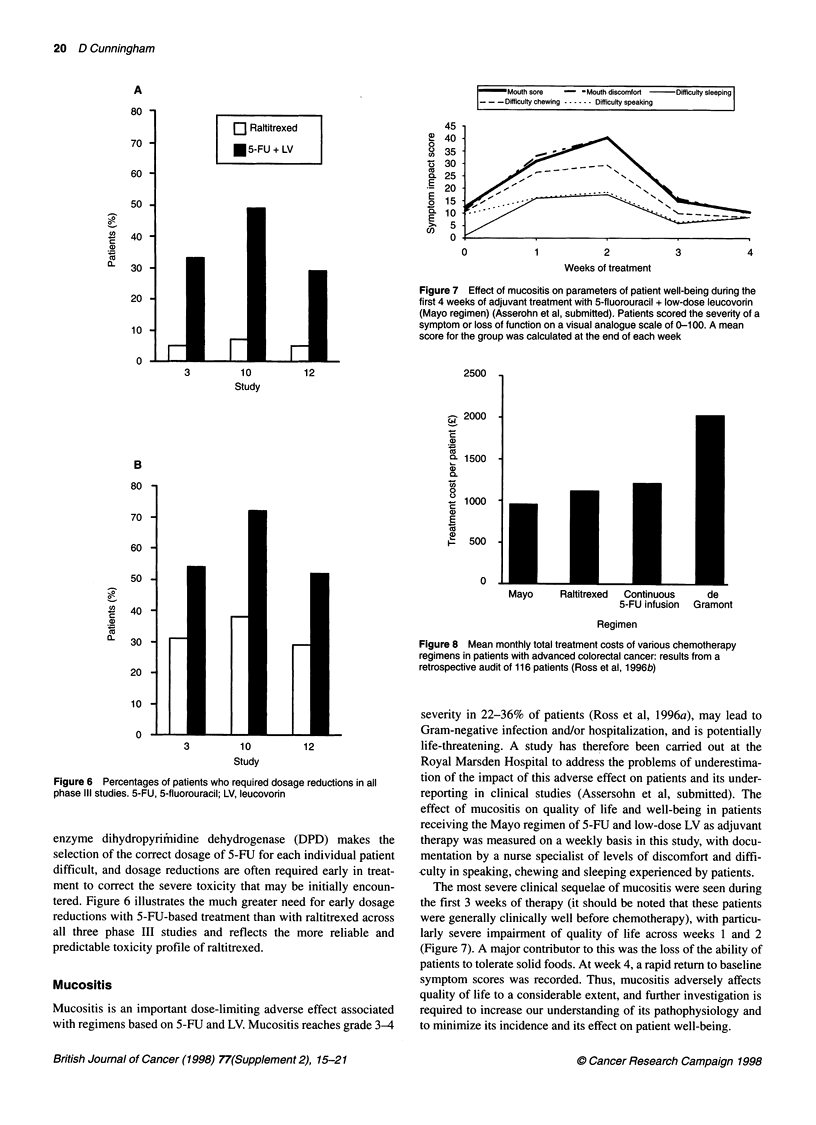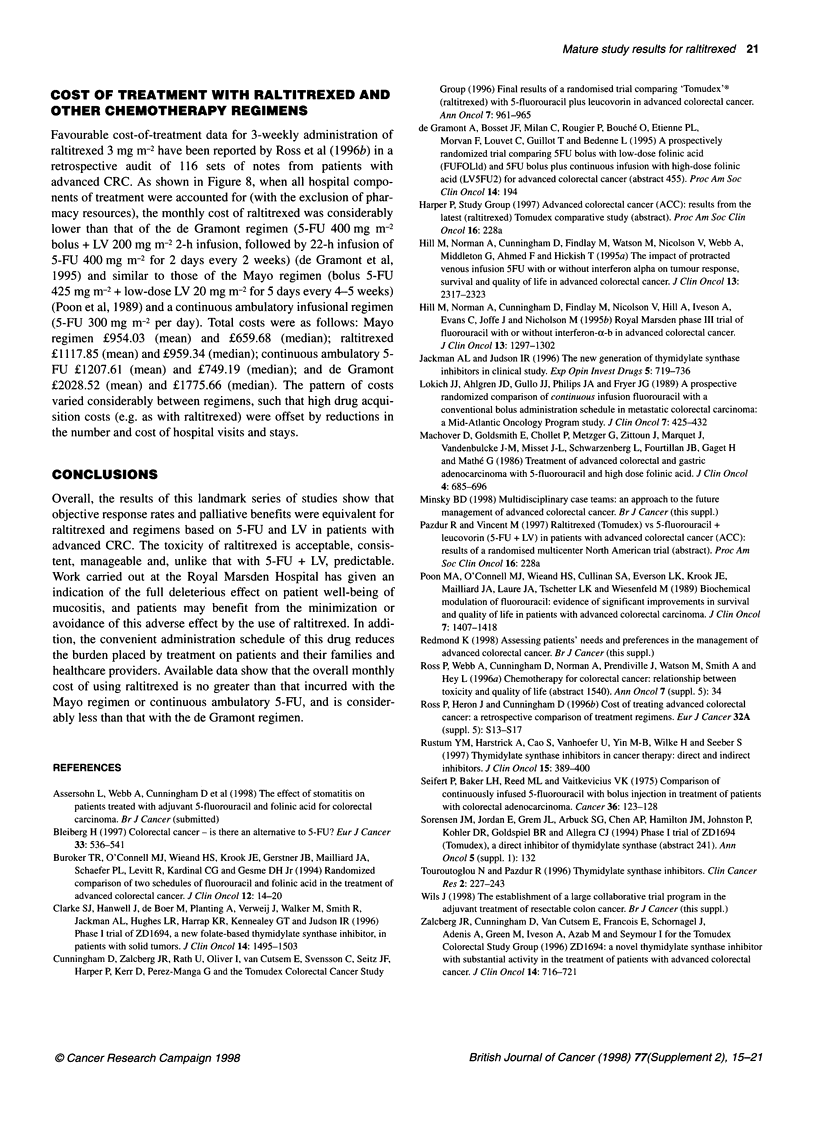# Mature results from three large controlled studies with raltitrexed ('Tomudex').

**DOI:** 10.1038/bjc.1998.421

**Published:** 1998

**Authors:** D. Cunningham

**Affiliations:** The Royal Marsden Hospital, Sutton, UK.

## Abstract

Since the publication of the results of phase I dose-finding studies, an extensive phase II and III clinical study programme has been undertaken to study the clinical efficacy and tolerability of the quinazoline folate analogue raltitrexed ('Tomudex'), a novel direct and specific inhibitor of thymidylate synthase. Two international phase III trials, studies 3 and 12, have compared raltitrexed 3 mg m(-2) with 5-fluorouracil (5-FU) plus low-dose leucovorin (LV) (Mayo regimen) or high-dose LV (Machover regimen) respectively. A North American study (study 10) was originally set up to compare two raltitrexed dosage arms (3.0 and 4.0 mg m[-2]) with 5-FU and low-dose LV, but the 4.0 mg m(-2) arm was discontinued prematurely because of excessive toxicity. Minimum follow-up times for studies 3, 10 and 12 were 15.5, 12 and 9 months, respectively (for data other than survival), with corresponding survival follow-up times of 26, 12 and 17 months. Objective response rates were similar for raltitrexed and 5-FU + LV, and palliative improvements were seen to a similar extent with both treatments in all phase III studies. Survival was statistically similar for raltitrexed and 5-FU + LV in both studies 3 and 12. Raltitrexed was, however, associated with inferior survival to 5-FU + low-dose LV in study 10, but there appears to be evidence that this was linked to an unconscious effect on investigator behaviour of early toxicity problems in this trial, in that patients appeared to be withdrawn from raltitrexed treatment without progression or protocolled toxicity. Moreover, it appeared that 5-FU + LV patients were continued on treatment after disease progression. 5-FU-based therapy was associated with a higher incidence of mucositis than raltitrexed in all studies, with the attainment of statistical significance in studies 3 and 12. Elevations in hepatic transaminase levels were seen with raltitrexed, but these are thought to be of no clinical significance. Overall, much greater levels of toxicity were seen with 5-FU + LV than with raltitrexed in early treatment cycles. In addition, retrospective UK audit data have shown the monthly cost of raltitrexed therapy to be similar to that of Mayo and continuous infusion 5-FU regimens, and appreciably lower than that of the de Gramont regimen of 5-FU (bolus + 22-h infusion) + high-dose LV. Thus, raltitrexed is an effective alternative to 5-FU-based therapy in patients with advanced colorectal cancer, with an acceptable and, unlike 5-FU, predictable toxicity profile. In particular, patients receiving raltitrexed may benefit from the minimization or avoidance of mucositis, and both patients and healthcare providers may find the convenient administration schedule of the drug advantageous.


British Journal of Cancer (1998) 77(Supplement 2), 15–21
© 1998 Cancer Research Campaign

### Mature results from three large controlled studies with raltitrexed ('Tomudex')

**D Cunningham**

The Royal Marsden Hospital, Sutton, UK

**Summary** Since the publication of the results of phase I dose-finding studies, an extensive phase II and III clinical study programme has been undertaken to study the clinical efficacy and tolerability of the quinazoline folate analogue raltitrexed ('Tomudex'), a novel direct and specific inhibitor of thymidylate synthase. Two international phase III trials, studies 3 and 12, have compared raltitrexed 3 mg $m^{-2}$ with 5-fluorouracil (5-FU) plus low-dose leucovorin (LV) (Mayo regimen) or high-dose LV (Machover regimen) respectively. A North American study (study 10) was originally set up to compare two raltitrexed dosage arms (3.0 and 4.0 mg $m^{-2}$) with 5-FU and low-dose LV, but the 4.0 mg $m^{-2}$ arm was discontinued prematurely because of excessive toxicity. Minimum follow-up times for studies 3, 10 and 12 were 15.5, 12 and 9 months, respectively (for data other than survival), with corresponding survival follow-up times of 26, 12 and 17 months. Objective response rates were similar for raltitrexed and 5-FU + LV, and palliative improvements were seen to a similar extent with both treatments in all phase III studies. Survival was statistically similar for raltitrexed and 5-FU + LV in both studies 3 and 12. Raltitrexed was, however, associated with inferior survival to 5-FU + low-dose LV in study 10, but there appears to be evidence that this was linked to an unconscious effect on investigator behaviour of early toxicity problems in this trial, in that patients appeared to be withdrawn from raltitrexed treatment without progression or protocolled toxicity. Moreover, it appeared that 5-FU + LV patients were continued on treatment after disease progression. 5-FU-based therapy was associated with a higher incidence of mucositis than raltitrexed in all studies, with the attainment of statistical significance in studies 3 and 12. Elevations in hepatic transaminase levels were seen with raltitrexed, but these are thought to be of no clinical significance. Overall, much greater levels of toxicity were seen with 5-FU + LV than with raltitrexed in early treatment cycles. In addition, retrospective UK audit data have shown the monthly cost of raltitrexed therapy to be similar to that of Mayo and continuous infusion 5-FU regimens, and appreciably lower than that of the de Gramont regimen of 5-FU (bolus + 22-h infusion) + high-dose LV. Thus, raltitrexed is an effective alternative to 5-FU-based therapy in patients with advanced colorectal cancer, with an acceptable and, unlike 5-FU, predictable toxicity profile. In particular, patients receiving raltitrexed may benefit from the minimization or avoidance of mucositis, and both patients and healthcare providers may find the convenient administration schedule of the drug advantageous.

**Keywords:** raltitrexed; Mayo regimen; Machover regimen; advanced colorectal cancer; phase III studies

Combinations of 5-fluorouracil (5-FU) and leucovorin (LV) have been regarded for some time as standard chemotherapy for advanced colorectal cancer (CRC) (Bleiberg, 1997). Although the optimal treatment regimen for these two drugs has not been unequivocally defined, the Mayo regimen of 5-FU and low-dose LV is widely regarded as an acceptable treatment and is the only regimen to have demonstrated a significant survival advantage over unmodulated 5-FU (Poon et al, 1989). However, other combination regimens, such as the Machover (5-FU with high-dose LV) regimen (Machover et al, 1986), or continuously infused 5-FU (Seifert et al, 1975; Lokich et al, 1989; de Gramont et al, 1995) are also used.

Despite their widespread acceptance, combinations of 5-FU and LV require complex and inconvenient dosing schedules and are associated with significant toxicity (Lokich et al, 1989; Poon et al, 1989; Buroker et al, 1994; Bleiberg, 1997). In view of the limitations of these therapies, there is a need for the development of new anti-cancer agents with greater or equivalent efficacy, improved tolerability and more acceptable administration schedules.

The quinazoline folate analogue raltitrexed ('Tomudex') was developed as part of an extensive research effort to produce a direct and specific thymidylate synthase inhibitor that lacks the non-specific effects on RNA and protein synthesis of 5-FU that are believed to contribute to the toxicity profile of this drug (Jackman and Judson, 1996; Touroutoglou and Pazdur, 1996; Bleiberg, 1997; Rustum et al, 1997). A simple dosage schedule of a single 15-min infusion once every 3 weeks is made possible with raltitrexed by the extensive polyglutamation and subsequent retention in tissues of the drug after its uptake into cells by a reduced folate carrier system. Phase I dose-ranging studies with raltitrexed (Sorensen et al, 1994; Clarke et al, 1996) indicated the maximum-tolerated doses of the drug to be 3.5–4.5 mg $m^{-2}$.

*Correspondence to:* D Cunningham, CRC Section of Medicine, Institute of Cancer Research, The Royal Marsden Hospital, Downs Road, Sutton SM2 5PR, UK

### PHASE II DATA ON RALTITREXED

In the phase II study (study 2C), conducted in 15 centres across Europe, Australia and South Africa, 177 patients with advanced CRC were recruited (of whom approximately 50% were patients of the Royal Marsden Hospital). Patients who had previously undergone chemotherapy were excluded (with the exception of the 5% of patients who had received prior adjuvant chemotherapy). Raltitrexed was given at a dosage of 3 mg $m^{-2}$ as a single 15-min intravenous infusion once every 3 weeks. An overall objective response rate (complete and partial remissions) of 26% (95% CI

'Tomudex' is a trademark, the property of Zeneca.

**Table 1** Demographic summary of studies 3, 10 and 12

| Study | Treatment | Mean age (years) | Male patients (%) | Colon/rectal tumour (%) | WHO performance status 0–1 at study entry (%) | Prior adjuvant chemotherapy (%) | Measurable disease (%) |
|---|---|---|---|---|---|---|---|
| 3 | Raltitrexed 3 mg m$^{-2}$ 15-min infusion 3-weekly | 61 | 60 | 59/40[a] | 90 | 5 | 96 |
| | 5-FU 425 mg m$^{-2}$ i.v. bolus + LV 20 mg m$^{-2}$ | 61 | 59 | 68/32 | 88 | 5 | 94 |
| 10 | Raltitrexed 3 mg m$^{-2}$ 15-min infusion 3-weekly | 61 | 62 | 79/20[a] | 90 | 13 | 93 |
| | 5-FU 425 mg m$^{-2}$ i.v. bolus + LV 20 mg m$^{-2}$ | 62 | 56 | 80/20 | 90 | 21 | 93 |
| 12 | Raltitrexed 3 mg m$^{-2}$ 15-min infusion 3-weekly | 60 | 62 | 65/35 | 90 | 10 | 94 |
| | 5-FU 400 mg m$^{-2}$ 15-min infusion + LV 200 mg m$^{-2}$ | 62 | 66 | 66/34 | 93 | 12 | 96 |

[a]One site unknown. 5-FU, 5-fluorouracil; LV, leucovorin.

**Table 2** Objective response rates in studies 3, 10 and 12 (percentages of patients)

| Response | Study 3 | | Study 10 | | Study 12 | |
|---|---|---|---|---|---|---|
| | Raltitrexed ($n$ = 223) | 5-FU+LDLV ($n$ = 216) | Raltitrexed ($n$ = 199) | 5-FU+LDLV ($n$ = 179) | Raltitrexed ($n$ = 230) | 5-FU+HDLV ($n$ = 222) |
| Complete (CR) | 3.6 | 3.7 | 2.8 | 1.4 | 3.2 | 3.6 |
| Partial (PR) | 15.7 | 13.0 | 11.5 | 13.8 | 15.4 | 14.5 |
| CR+PR | 19.3 | 16.7[a] | 14.3 | 15.2[b] | 18.6 | 18.1[c] |
| Stable disease | 35.0 | 32.4 | 33.2 | 40.0 | 51.4 | 52.4 |

[a]$P$ = 0.48 between groups; [b]$P$ = 0.597 between groups; [c]$P$ = 0.896 between groups. 5-FU, 5-fluorouracil; LDLV, low-dose leucovorin; HDLV, high-dose leucovorin.

**Table 3** Palliative benefits of chemotherapy in studies 3, 10 and 12 (percentages of patients)

| Parameter | Study 3 | | Study 10 | | Study 12 | |
|---|---|---|---|---|---|---|
| | Raltitrexed ($n$ = 223) | 5-FU+LDLV ($n$ = 216) | Raltitrexed ($n$ = 199) | 5-FU+LDLV ($n$ = 179) | Raltitrexed ($n$ = 230) | 5-FU+HDLV ($n$ = 222) |
| Weight gain ≥5% | 16.6 | 15.7 | 21.1 | 27.4 | 13.0 | 18.9 |
| Improvement in performance status | 36.4 | 29.7 | 39.1 | 40.8 | 38.2 | 31.1 |
| Improvement in disease symptoms | NA | NA | NA | NA | 86.1 | 83.1 |

5-FU, 5-fluorouracil; LDLV, low-dose leucovorin; HDLV, high-dose leucovorin; NA, not available.

19–33%) was reported, which compared favourably with response rates seen with optimally modulated 5-FU (Zalcberg et al, 1996). Median survival was 11.2 months (95% CI 9.6–13.1 months).

### PHASE III COMPARISONS OF RALTITREXED WITH 5-FLUOROURACIL AND LEUCOVORIN IN PATIENTS WITH ADVANCED COLORECTAL CANCER

After these encouraging data, three prospective randomized phase III clinical studies were set up to compare raltitrexed with modulated 5-FU in patients with advanced CRC (Cunningham et al, 1996; Harper, 1997; Pazdur and Vincent, 1997). Studies 3 and 12 were conducted in Europe, South Africa and Australasia, and study 10 in North America. In study 3 (439 patients), raltitrexed 3 mg m$^{-2}$, given as a 15-min infusion once every 3 weeks, was compared with the Mayo regimen of 5-FU (425 mg m$^{-2}$ bolus) plus low-dose LV (20 mg m$^{-2}$) daily for 5 days every 4–5 weeks. In study 12 (495 patients), the same dosage of raltitrexed was compared with the Machover regimen of 5-FU (400 mg m$^{-2}$ given as a 15-min infusion) plus high-dose LV (200 mg m$^{-2}$) daily for 5 days every 4 weeks.

A different approach was taken in the North American study 10, in which two raltitrexed treatment arms were compared with the Mayo regimen of 5-FU plus low-dose LV. In this study, raltitrexed was given at 3-weekly dosages of 3 and 4 mg m$^{-2}$, the higher

© Cancer Research Campaign 1998

**Figure 1** Percentages of patients categorized by response with (**A**) weight improvement of at least 5% and (**B**) improvement in World Health Organization (WHO) performance status (baseline performance status ≥ 1) during study 3. Figures under columns denote numbers of patients. 5-FU, 5-fluorouracil; CR, complete response; PR, partial response; SD, stable disease; LDLV, low-dose leucovorin dosage being selected on the basis of results of one of the phase I trials, conducted in the USA, that had suggested that raltitrexed might be given safely at this dosage level (Sorensen et al, 1994). These findings were in contrast to the other phase I study (which had been carried out in the UK and the Netherlands), in which a dosage of 3 mg $m^{-2}$ was recommended for future investigations (Clarke et al, 1996). However, the 4 mg $m^{-2}$ dosage arm had to be closed prematurely because of unacceptable toxicity, and the subsequent intention-to-treat analysis was based on the resulting two-arm study in 427 patients. Overall, this series of phase III studies has provided a database of results from nearly 1500 patients, which represents a unique resource.

Maturity of data in the three studies is as follows: minimum follow-up times for efficacy and safety for studies 3, 10 and 12 are 15.5, 12 and 9 months respectively. Corresponding times for survival follow-up are 26, 12 and 17 months. Demographic details for the patients enrolled are shown in Table 1 and show the treatment groups to be very similar in each study. Interestingly, there was an approximate doubling in the number of patients who had received previous adjuvant chemotherapy in study 12 compared with study 3. This reflects the rapid evolution of clinical practice in the management of CRC that followed the publication of new data on adjuvant chemotherapy between the times that these two studies were set up (Wils, 1998).

Median durations of therapy for each treatment group were very similar in study 3. Patients in the raltitrexed arm were treated for a median 15.2 weeks (five cycles), compared with 15.0 weeks (three cycles) for patients who received 5-FU + LV. In study 12, median duration of treatment was shorter for raltitrexed (12.7 weeks; four cycles) than for 5-FU + LV (16.9 weeks; four cycles). However, in study 10, there was a marked difference between median treatment durations: 12.1 weeks (four cycles) for raltitrexed and 22.3 weeks (five cycles) for 5-FU + LV (discussed later).

### Objective response rates

Objective response was similar between treatments across all phase III clinical trials (Table 2). The high proportion of patients recorded as having stable disease in study 12 can be attributed to the early first assessment (6 weeks) in this study relative to studies 3 and 10 (12 weeks), and illustrates how the changing of assessment times can affect the results obtained in a clinical trial.

**Table 4** Survival data from phase II and III clinical studies of raltitrexed

| Study | Therapy | Median survival (months) | Deaths during follow-up (% of patients) | Hazard ratio (95% CI) | *P* (between treatments) |
|---|---|---|---|---|---|
| 2C | Raltitrexed | 11.2 | 77 | NA | NA |
| 3 | Raltitrexed | 10.1 | 89 | 1.09 (0.89–1.33) | 0.42 |
| | 5-FU+LDLV | 10.2 | 85 | | |
| 10 | Raltitrexed | 9.7 | 75 | 1.35 (1.07–1.71) | 0.01 |
| | 5-FU+LDLV | 12.7 | 65 | | |
| 12 | Raltitrexed | 10.9 | 75 | 1.15 (0.93–1.42) | 0.197 |
| | 5-FU+HDLV | 12.3 | 69 | | |

5-FU, 5-fluorouracil; LDLV, low-dose leucovorin; HDLV, high-dose leucovorin; NA, not applicable.

© Cancer Research Campaign 1998

**Figure 2** Kaplan–Meier survival curves showing probability of survival over time for raltitrexed and 5-fluorouracil (5-FU) + high-dose leucovorin (HDLV) in phase III study 12

**Figure 3** Survival in advanced colorectal cancer: prospective data collected at the Royal Marsden hospital. Protracted intravenous infusion (PVI) of 5-fluorouracil (5-FU) was administered at a dosage of 300 mg $m^{-2}$ per day (Hill et al, 1995*a*). The Wadler schedule of bolus 5-FU was used (750 mg $m^{-2}$ per day by continuous infusion for 5 days, followed by bolus 5-FU 750 mg $m^{-2}$ once weekly from the start of week 2) (Hill et al, 1995*b*). Raltitrexed 3 mg $m^{-2}$ was given by 15-min intravenous infusion every 3 weeks

### Palliative benefits of treatment with raltitrexed

There is an increasing appreciation of the importance of the palliative effects of treatment in patients with advanced cancer; Redmond (1998) and Minsky (1998) have shown how the overall effectiveness of treatment can be enhanced by using therapies that combine increased well-being for patients with clinical efficacy in terms of objective response and survival. In the present phase III clinical trials, clinically significant palliative benefits were obtained to a similar extent with raltitrexed and 5-FU + LV in terms of weight gain, improvements in performance status and reductions in disease symptoms (e.g. anorexia, lethargy) (Table 3). In study 12, more than 80% of patients with symptoms at study entry reported improvements after treatment with either drug, which indicates the clinical significance of the quality-of-life benefits that can be obtained with such treatments. Palliative improvements were more frequent in patients who responded to treatment or who had stabilization of disease than in patients whose disease progressed (Figure 1). These data suggest that significant clinical benefit is obtained not only in patients who respond to chemotherapy, but also in those who achieve disease stabilization. They also concur with observations in everyday clinical practice that indicate that many patients feel better when their disease is stabilized.

**Figure 4** Pooled survival data from 43 clinical studies in patients with advanced colorectal cancer. 5-FU, 5-fluorouracil; HDLV, high-dose leucovorin; LDLV, low-dose leucovorin

© Cancer Research Campaign 1998

**Table 5** WHO grade 3 or 4 adverse events that were observed in at least 5% of patients in studies 3, 10 and 12

| Adverse event | Study 3 | | Study 10 | | Study 12 | |
|---|---|---|---|---|---|---|
| | Raltitrexed ($n$ = 223) | 5-FU+LDLV ($n$ = 216) | Raltitrexed ($n$ = 199) | 5-FU+LDLV ($n$ = 179) | Raltitrexed ($n$ = 230) | 5-FU+HDLV ($n$ = 222) |
| Leucopenia | 14 | 30[a] | 18 | 41[a] | 6 | 13 |
| Mucositis (stomatitis) | 2 | 22[a] | 3 | 10 | 2 | 16[a] |
| Anaemia | 9[a] | 2 | 9 | 4 | 5 | 2 |
| Elevated transaminases | 10[a] | 0 | 7 | 1 | 13[a] | 9 |
| Nausea/vomiting | 13 | 9 | 13 | 8 | 9 | 9 |
| Asthenia (severe) | 6 | 2 | 18 | 10 | 5 | 2 |
| Diarrhoea | 14 | 14 | 10 | 13 | 10 | 19 |
| Thrombocytopenia | 4 | 1 | 5 | 3 | 3 | 0 |
| Infection | 5 | 5 | 6 | 7 | 4 | 3 |
| Pain | 5 | 7 | 14 | 16 | 5 | 4 |

[a]$P < 0.001$ (Holm's procedure).

**Figure 5** Percentages of patients with WHO grade 3 or 4 adverse events who required dosage reduction in study 3. 5-FU, 5-fluorouracil; LDLV, low-dose leucovorin

### Survival

Survival is a key end point in randomized clinical studies of anti-cancer agents, and data are available for all such studies conducted to date with raltitrexed (Table 4). The median survival of 11.2 months obtained in the phase II trial was of the order expected in a patient sample of the type recruited, and data from the first phase III study (study 3) showed the survival curves for raltitrexed and 5-FU + LV to be superimposable. Survival data from study 10 (North American) showed a significantly shorter survival with raltitrexed than with the Mayo regimen of 5-FU + LV (Table 4). It is thought that this was attributable to the much longer duration of treatment of 5-FU + LV patients, which itself appears to be a consequence of unconscious investigator bias. Data indicate that patients were withdrawn early from raltitrexed treatment without apparently meeting the protocol requirements for progression or toxicity, and that proportionately more 5-FU + LV patients were continued on treatment after disease progression. These behaviours probably stem from the termination of the 4 mg $m^{-2}$ raltitrexed arm in this trial because of unacceptable toxicity, coupled with the familiarity of the investigators with the 5-FU + LV regimen. There was no statistically significant difference between treatments in study 12 (illustrated in Figure 2). Non-randomized prospective phase II data collected at the Royal Marsden Hospital also show comparability of survival with raltitrexed and different 5-FU regimens (Figure 3).

An analysis of 43 prospective, randomized, peer-reviewed clinical studies of chemotherapy in advanced CRC (published in English-language journals since 1991, in addition to key studies published before this date) has shown consistency of survival results across phase II and III trials of raltitrexed compared with those obtained with other regimens. These disparities are illustrated in Figure 4 and may be attributed to variable study conditions at different centres or to differences in patient selection criteria and/or methods. In particular, there is a wide variation across the literature in survival results with the de Gramont 5-FU + high-dose LV regimen. Notably, however, there are evident advantages relative to best supportive care alone in the use of chemotherapy in advanced CRC: median survival times with raltitrexed, for example, are approximately twice those seen with best supportive care alone.

### ADVERSE EVENTS IN PHASE III STUDIES

A summary of WHO grade 3 and 4 adverse events that were recorded in at least 5% of patients in the phase III studies is given in Table 5. Higher frequencies of mucositis were seen with 5-FU-based regimens in all trials, with statistical significance being attained in studies 3 and 12. Higher frequencies of disturbance of hepatic enzymes were seen with raltitrexed (7–13%). However, many antimetabolites (e.g. methotrexate) are associated with transient and reversible increases in hepatic transaminase levels that are not thought to have any clinical significance. The incidence of mucositis was lower for both treatments in study 10; this may be attributable to the more widespread use of 'ice-chip' therapy (i.e. the retention of crushed ice cubes in the mouth) in the USA. 5-FU + LV was also associated with a significantly higher incidence of leucopenia in studies 3 and 10 (with a trend towards a higher incidence in study 12). Otherwise, tolerability of raltitrexed and 5-FU-based therapy was broadly similar across all studies. Examination of the incidence of WHO grade 3 or 4 adverse events by treatment cycle revealed that early treatment cycles were associated with much greater toxicity with 5-FU + LV than with raltitrexed. This high incidence of toxicity decreased with each cycle until the incidence of grade 3–4 adverse events was statistically similar between treatment groups from cycle 4 onwards (Figure 5). High early levels of toxicity in patients receiving 5-FU + LV are associated with the variation across populations in the ability of patients to metabolize this drug. Variable activity of the degradative

© Cancer Research Campaign 1998

**Figure 6** Percentages of patients who required dosage reductions in all phase III studies. 5-FU, 5-fluorouracil; LV, leucovorin

**Figure 7** Effect of mucositis on parameters of patient well-being during the first 4 weeks of adjuvant treatment with 5-fluorouracil + low-dose leucovorin (Mayo regimen) (Asserohn et al, submitted). Patients scored the severity of a symptom or loss of function on a visual analogue scale of 0–100. A mean score for the group was calculated at the end of each week

**Figure 8** Mean monthly total treatment costs of various chemotherapy regimens in patients with advanced colorectal cancer: results from a retrospective audit of 116 patients (Ross et al, 1996*b*)

enzyme dihydropyrimidine dehydrogenase (DPD) makes the selection of the correct dosage of 5-FU for each individual patient difficult, and dosage reductions are often required early in treatment to correct the severe toxicity that may be initially encountered. Figure 6 illustrates the much greater need for early dosage reductions with 5-FU-based treatment than with raltitrexed across all three phase III studies and reflects the more reliable and predictable toxicity profile of raltitrexed.

### Mucositis

Mucositis is an important dose-limiting adverse effect associated with regimens based on 5-FU and LV. Mucositis reaches grade 3–4 severity in 22–36% of patients (Ross et al, 1996*a*), may lead to Gram-negative infection and/or hospitalization, and is potentially life-threatening. A study has therefore been carried out at the Royal Marsden Hospital to address the problems of underestimation of the impact of this adverse effect on patients and its under-reporting in clinical studies (Assersohn et al, submitted). The effect of mucositis on quality of life and well-being in patients receiving the Mayo regimen of 5-FU and low-dose LV as adjuvant therapy was measured on a weekly basis in this study, with documentation by a nurse specialist of levels of discomfort and difficulty in speaking, chewing and sleeping experienced by patients.

The most severe clinical sequelae of mucositis were seen during the first 3 weeks of therapy (it should be noted that these patients were generally clinically well before chemotherapy), with particularly severe impairment of quality of life across weeks 1 and 2 (Figure 7). A major contributor to this was the loss of the ability of patients to tolerate solid foods. At week 4, a rapid return to baseline symptom scores was recorded. Thus, mucositis adversely affects quality of life to a considerable extent, and further investigation is required to increase our understanding of its pathophysiology and to minimize its incidence and its effect on patient well-being.

© Cancer Research Campaign 1998

### COST OF TREATMENT WITH RALTITREXED AND OTHER CHEMOTHERAPY REGIMENS

Favourable cost-of-treatment data for 3-weekly administration of raltitrexed 3 mg $m^{-2}$ have been reported by Ross et al (1996*b*) in a retrospective audit of 116 sets of notes from patients with advanced CRC. As shown in Figure 8, when all hospital components of treatment were accounted for (with the exclusion of pharmacy resources), the monthly cost of raltitrexed was considerably lower than that of the de Gramont regimen (5-FU 400 mg $m^{-2}$ bolus + LV 200 mg $m^{-2}$ 2-h infusion, followed by 22-h infusion of 5-FU 400 mg $m^{-2}$ for 2 days every 2 weeks) (de Gramont et al, 1995) and similar to those of the Mayo regimen (bolus 5-FU 425 mg $m^{-2}$ + low-dose LV 20 mg $m^{-2}$ for 5 days every 4–5 weeks) (Poon et al, 1989) and a continuous ambulatory infusional regimen (5-FU 300 mg $m^{-2}$ per day). Total costs were as follows: Mayo regimen £954.03 (mean) and £659.68 (median); raltitrexed £1117.85 (mean) and £959.34 (median); continuous ambulatory 5-FU £1207.61 (mean) and £749.19 (median); and de Gramont £2028.52 (mean) and £1775.66 (median). The pattern of costs varied considerably between regimens, such that high drug acquisition costs (e.g. as with raltitrexed) were offset by reductions in the number and cost of hospital visits and stays.

### CONCLUSIONS

Overall, the results of this landmark series of studies show that objective response rates and palliative benefits were equivalent for raltitrexed and regimens based on 5-FU and LV in patients with advanced CRC. The toxicity of raltitrexed is acceptable, consistent, manageable and, unlike that with 5-FU + LV, predictable. Work carried out at the Royal Marsden Hospital has given an indication of the full deleterious effect on patient well-being of mucositis, and patients may benefit from the minimization or avoidance of this adverse effect by the use of raltitrexed. In addition, the convenient administration schedule of this drug reduces the burden placed by treatment on patients and their families and healthcare providers. Available data show that the overall monthly cost of using raltitrexed is no greater than that incurred with the Mayo regimen or continuous ambulatory 5-FU, and is considerably less than that with the de Gramont regimen.

### REFERENCES

Assersohn L, Webb A, Cunningham D et al (1998) The effect of stomatitis on patients treated with adjuvant 5-fluorouracil and folinic acid for colorectal carcinoma. *Br J Cancer* (submitted)

Bleiberg H (1997) Colorectal cancer – is there an alternative to 5-FU? *Eur J Cancer* **33**: 536–541

Buroker TR, O'Connell MJ, Wieand HS, Krook JE, Gerstner JB, Mailliard JA, Schaefer PL, Levitt R, Kardinal CG and Gesme DH Jr (1994) Randomized comparison of two schedules of fluorouracil and folinic acid in the treatment of advanced colorectal cancer. *J Clin Oncol* **12**: 14–20

Clarke SJ, Hanwell J, de Boer M, Planting A, Verweij J, Walker M, Smith R, Jackman AL, Hughes LR, Harrap KR, Kennealey GT and Judson IR (1996) Phase I trial of ZD1694, a new folate-based thymidylate synthase inhibitor, in patients with solid tumors. *J Clin Oncol* **14**: 1495–1503

Cunningham D, Zalcberg JR, Rath U, Oliver I, van Cutsem E, Svensson C, Seitz JF, Harper P, Kerr D, Perez-Manga G and the Tomudex Colorectal Cancer Study Group (1996) Final results of a randomised trial comparing 'Tomudex'® (raltitrexed) with 5-fluorouracil plus leucovorin in advanced colorectal cancer. *Ann Oncol* **7**: 961–965

de Gramont A, Bosset JF, Milan C, Rougier P, Bouché O, Etienne PL, Morvan F, Louvet C, Guillot T and Bedenne L (1995) A prospectively randomized trial comparing 5FU bolus with low-dose folinic acid (FUFOLld) and 5FU bolus plus continuous infusion with high-dose folinic acid (LV5FU2) for advanced colorectal cancer (abstract 455). *Proc Am Soc Clin Oncol* **14**: 194

Harper P, Study Group (1997) Advanced colorectal cancer (ACC): results from the latest (raltitrexed) Tomudex comparative study (abstract). *Proc Am Soc Clin Oncol* **16**: 228a

Hill M, Norman A, Cunningham D, Findlay M, Watson M, Nicolson V, Webb A, Middleton G, Ahmed F and Hickish T (1995*a*) The impact of protracted venous infusion 5FU with or without interferon alpha on tumour response, survival and quality of life in advanced colorectal cancer. *J Clin Oncol* **13**: 2317–2323

Hill M, Norman A, Cunningham D, Findlay M, Nicolson V, Hill A, Iveson A, Evans C, Joffe J and Nicholson M (1995*b*) Royal Marsden phase III trial of fluorouracil with or without interferon-α-b in advanced colorectal cancer. *J Clin Oncol* **13**: 1297–1302

Jackman AL and Judson IR (1996) The new generation of thymidylate synthase inhibitors in clinical study. *Exp Opin Invest Drugs* **5**: 719–736

Lokich JJ, Ahlgren JD, Gullo JJ, Philips JA and Fryer JG (1989) A prospective randomized comparison of *continuous* infusion fluorouracil with a conventional bolus administration schedule in metastatic colorectal carcinoma: a Mid-Atlantic Oncology Program study. *J Clin Oncol* **7**: 425–432

Machover D, Goldsmith E, Chollet P, Metzger G, Zittoun J, Marquet J, Vandenbulcke J-M, Misset J-L, Schwarzenberg L, Fourtillan JB, Gaget H and Mathé G (1986) Treatment of advanced colorectal and gastric adenocarcinoma with 5-fluorouracil and high dose folinic acid. *J Clin Oncol* **4**: 685–696

Minsky BD (1998) Multidisciplinary case teams: an approach to the future management of advanced colorectal cancer. *Br J Cancer* (this suppl.)

Pazdur R and Vincent M (1997) Raltitrexed (Tomudex) vs 5-fluorouracil + leucovorin (5-FU + LV) in patients with advanced colorectal cancer (ACC): results of a randomised multicenter North American trial (abstract). *Proc Am Soc Clin Oncol* **16**: 228a

Poon MA, O'Connell MJ, Wieand HS, Cullinan SA, Everson LK, Krook JE, Mailliard JA, Laure JA, Tschetter LK and Wiesenfeld M (1989) Biochemical modulation of fluorouracil: evidence of significant improvements in survival and quality of life in patients with advanced colorectal carcinoma. *J Clin Oncol* **7**: 1407–1418

Redmond K (1998) Assessing patients' needs and preferences in the management of advanced colorectal cancer. *Br J Cancer* (this suppl.)

Ross P, Webb A, Cunningham D, Norman A, Prendiville J, Watson M, Smith A and Hey L (1996*a*) Chemotherapy for colorectal cancer: relationship between toxicity and quality of life (abstract 1540). *Ann Oncol* **7** (suppl. 5): 34

Ross P, Heron J and Cunningham D (1996*b*) Cost of treating advanced colorectal cancer: a retrospective comparison of treatment regimens. *Eur J Cancer* **32A** (suppl. 5): S13–S17

Rustum YM, Harstrick A, Cao S, Vanhoefer U, Yin M-B, Wilke H and Seeber S (1997) Thymidylate synthase inhibitors in cancer therapy: direct and indirect inhibitors. *J Clin Oncol* **15**: 389–400

Seifert P, Baker LH, Reed ML and Vaitkevicius VK (1975) Comparison of continuously infused 5-fluorouracil with bolus injection in treatment of patients with colorectal adenocarcinoma. *Cancer* **36**: 123–128

Sorensen JM, Jordan E, Grem JL, Arbuck SG, Chen AP, Hamilton JM, Johnston P, Kohler DR, Goldspiel BR and Allegra CJ (1994) Phase I trial of ZD1694 (Tomudex), a direct inhibitor of thymidylate synthase (abstract 241). *Ann Oncol* **5** (suppl. 1): 132

Touroutoglou N and Pazdur R (1996) Thymidylate synthase inhibitors. *Clin Cancer Res* **2**: 227–243

Wils J (1998) The establishment of a large collaborative trial program in the adjuvant treatment of resectable colon cancer. *Br J Cancer* (this suppl.)

Zalcberg JR, Cunningham D, Van Cutsem E, Francois E, Schornagel J, Adenis A, Green M, Iveson A, Azab M and Seymour I for the Tomudex Colorectal Study Group (1996) ZD1694: a novel thymidylate synthase inhibitor with substantial activity in the treatment of patients with advanced colorectal cancer. *J Clin Oncol* **14**: 716–721

© Cancer Research Campaign 1998